# Adjuvant chemotherapy versus chemoradiotherapy for small cell lung cancer with lymph node metastasis: a retrospective observational study with use of a national database in Japan

**DOI:** 10.1186/s12885-017-3610-0

**Published:** 2017-09-02

**Authors:** Hirokazu Urushiyama, Taisuke Jo, Hideo Yasunaga, Yasuhiro Yamauchi, Hiroki Matsui, Wakae Hasegawa, Hideyuki Takeshima, Yoshihisa Hiraishi, Akihisa Mitani, Kiyohide Fushimi, Takahide Nagase

**Affiliations:** 1 Department of Respiratory Medicine, Graduate School of Medicine, The University of Tokyo, 7-3-1 Hongo, Bunkyo-ku, Tokyo, 113-8655 Japan; 2 Department of Clinical Epidemiology and Health Economics, School of Public Health, The University of Tokyo, 7-3-1 Hongo, Bunkyo-ku, Tokyo, 113-8655 Japan; 3 Department of Health Policy and Informatics, Graduate School of Medicine, Tokyo Medical and Dental University, 1-5-45 Yushima, Bunkyo-ku, Tokyo, 113-8510 Japan

**Keywords:** Small cell lung cancer, Postoperative therapy, Adjuvant chemotherapy, Adjuvant chemoradiotherapy, Recurrence-free survival, Clinical epidemiology

## Abstract

**Background:**

The optimal postoperative treatment strategy for small cell lung cancer (SCLC) remains unclear, especially in patients with lymph node metastasis. We aimed to compare the outcomes of patients with SCLC and lymph node metastasis treated with postoperative adjuvant chemotherapy or chemoradiotherapy.

**Methods:**

We retrospectively collected data on patients with postoperative SCLC diagnosed with N1 and N2 lymph node metastasis from the Diagnosis Procedure Combination database in Japan, between July 2010 and March 2015. We extracted data on patient age, sex, comorbidities, and TNM classification at lung surgery; operative procedures, chemotherapy drugs, and radiotherapy during hospitalization; and discharge status. Recurrence-free survival was compared between the chemotherapy and chemoradiotherapy groups using multivariable Cox regression analysis.

**Results:**

Median recurrence-free survival was 1146 days (95% confidence interval [CI], 885–1407) in the chemotherapy group (*n* = 489) and 873 days (95% CI, 464–1282) in the chemoradiotherapy group (*n* = 75). There was no significant difference between these after adjusting for patient backgrounds (hazard ratio, 1.29; 95% CI, 0.91–1.84).

**Conclusions:**

There was no significant difference in recurrence-free survival between patients with SCLC and N1-2 lymph node metastasis treated with postoperative adjuvant chemotherapy and chemoradiotherapy. Further randomized clinical trials are needed to address this issue.

## Background

Small cell lung cancer (SCLC) comprises 10%–20% of all lung cancers [1]. However, surgery is not appropriate in most SCLC patients, and only about 5% of SCLCs are considered to be surgically resectable [2]. In the case of postoperative SCLC with regional lymph node metastasis, European Society for Medical Oncology guidelines [3] and Japanese consensus treatment guidelines recommend adjuvant chemotherapy, with adjuvant chemoradiotherapy as an alternative option, while National Comprehensive Cancer Network guidelines recommend adjuvant chemoradiotherapy, but also note the lack of any data to support this recommendation [4]. The rarity of resectable SCLC means that, to the best of our knowledge, no prospective studies have compared postoperative adjuvant chemotherapy and chemoradiotherapy in patients with SCLC, and the optimal treatment thus remains unclear, especially in patients with regional lymph node metastasis.

The aim of this study was to compare the prognosis of patients with SCLC diagnosed with regional lymph node metastasis treated with postoperative adjuvant chemotherapy or chemoradiotherapy, using information from a national inpatient database in Japan.

## Methods

### Data source

The Diagnosis Procedure Combination (DPC) database [5] is a national inpatient database in Japan, covering approximately 50% of acute-care inpatients. It includes data on the following: patient age, sex, body height and weight (body mass index), primary diagnosis, TNM classification, Charlson comorbidity index, and Barthel index on admission; operative procedures, chemotherapy drugs, and radiotherapy during hospitalization; and discharge status. This study was approved by the Institutional Review Board of The University of Tokyo. The board waived the requirement for informed patient consent because of the anonymous nature of the data.

### Patient selection

We retrospectively collected data for patients with SCLC defined by the International Statistical Classification of Diseases and Related Health Problems-10th revision (ICD-10), who underwent surgery to remove one or more lung lobe(s) because of malignant lung cancer, between July 2010 and March 2015. We excluded patients aged ≤17 years and patients with a history of other cancers at the time of lung surgery. We selected patients who were diagnosed with N1–2 lymph node metastasis at their first adjuvant chemotherapy, which included agents such as cisplatin, carboplatin, etoposide, or irinotecan, within 3 months after surgery. We further selected patients who received radiotherapy for ≥17 consecutive days within 5 months after surgery. Patients who received both chemotherapy and radiotherapy were defined as the adjuvant chemoradiotherapy group, and patients who received chemotherapy alone were defined as the adjuvant chemotherapy group.

### Primary outcome

The primary outcome of this study was recurrence-free survival, defined as the time to any first event including relapse or death. Relapse was defined as: gamma-knife therapy starting >3 months after surgery; radiotherapy starting >9 months after surgery; diagnosis of distant metastasis (ICD-10 codes, C40, C41, C71, C72, C77, C787, C793 and C797); chemotherapy with the above-mentioned four drugs after >9 months; and chemotherapy with topotecan or amrubicin.

### Statistical analysis

Patient characteristics were compared between the groups using χ^2^ tests. Survival curves were constructed using the Kaplan–Meier method and compared between the groups using log-rank tests. Recurrence-free survival was compared between the groups by multivariable Cox regression analysis, after adjusting for patient backgrounds. The threshold for significance was *P* < 0.05. All statistical analyses were performed using SPSS version 22.0 (IBM SPSS Inc., Armonk, NY, USA).

## Results

### Patient characteristics

We identified 564 patients with SCLC who underwent lung resection followed by adjuvant chemotherapy (*n* = 489) or chemoradiotherapy (*n* = 75) during the study period. Patient characteristics on admission for primary chemotherapy after lung surgery are shown in Table 1. The proportion of women was higher in the chemotherapy than in the chemoradiotherapy group (*P* = 0.038), but there were no significant differences between the groups in any other patient characteristics.

**Table 1:** Patient characteristics

	Chemotherapy group		Chemoradiotherapy group		
	(*n* = 489)		(*n* = 75)		*P*
Cancer staging					
N factor					0.12
N1	243	(49.7)	30	(40.0)	
N2	246	(50.3)	45	(60.0)	
T factor					0.31
T0–1	203	(41.5)	37	(49.3)	
T2	192	(39.3)	21	(28.0)	
T3–4	90	(18.4)	16	(21.3)	
Tx	4	(0.8)	1	(1.3)	
Age (years)					0.97
18–49	24	(4.9)	3	(4.0)	
50–64	149	(30.5)	22	(29.3)	
65–74	242	(49.5)	39	(52.0)	
≥ 75	74	(15.1)	11	(14.7)	
Sex					0.038
Male	379	(77.5)	66	(88.0)	
Female	110	(22.5)	9	(12.0)	
Body mass index (kg/m^2^)					0.83
< 18.5	33	(6.8)	7	(9.3)	
18.5–24.9	325	(66.5)	48	(64.0)	
≥ 25	127	(26.0)	19	(25.3)	
Missing	4	(0.8)	1	(1.3)	
Charlson comorbidity index					0.32
0–2	316	(64.6)	44	(58.7)	
≥ 3	173	(35.4)	31	(41.3)	
Activity of daily life (Barthel index)					0.99
Independent (100–95)	465	(95.1)	71	(94.5)	
Dependent (≤90)	12	(2.5)	2	(2.7)	
Missing	12	(2.5)	2	(2.7)	

Data expressed as number (%) in each group

The details of adjuvant chemotherapy are shown in Table 2. The proportions of patients who received more than three cycles of adjuvant chemotherapy were 60.8% in the chemotherapy group and 72.0% in the chemoradiotherapy group. The numbers of cycles of cisplatin or carboplatin were consistent with the total number of courses. Totals of 181 patients in the chemotherapy group and six in the chemoradiotherapy group received neither etoposide nor irinotecan.

**Table 2:** Adjuvant chemotherapy

	Chemotherapy group (*n* = 489)		Chemoradiotherapy group (*n* = 75)	
Total course (cycles)				
1–2	192	(39.3)	21	(28.0)
3–4	256	(52.4)	45	(60.0)
≥ 5	41	(8.4)	9	(12.0)
Drug (cycles)				
Cisplatin or carboplatin				
0	0	0.0	2	(2.7)
1–2	197	(40.3)	21	(28.0)
3–4	258	(52.8)	44	(58.7)
≥ 5	34	(7.0)	8	(10.7)
Etoposide				
0	254	(51.9)	18	(24.0)
1–2	84	(17.2)	14	(18.7)
3–4	132	(27.0)	38	(50.7)
≥ 5	19	(3.9)	5	(6.7)
Irinotecan				
0	416	(85.1)	63	(84.0)
1–2	31	(6.3)	7	(9.3)
3–4	38	(7.8)	3	(4.0)
≥ 5	4	(0.8)	2	(2.7)

Data expressed as number (%) in each group

### Recurrence-free survival

Median recurrence-free survival was 1146 days (95% confidence interval [CI], 885–1407) in the chemotherapy group, 873 days (464–1282) in the chemoradiotherapy group, and 1120 days (915–1325) in all patients. The equivalent median recurrence-free survivals in N1 patients were 1484 days (not predictable), 974 days (not predictable), and 1158 days (756–1560), respectively, and in N2 patients were 1120 days (831–1409), 596 days (163–1029), and 983 days (718–1248), respectively.

**Fig. 1:**
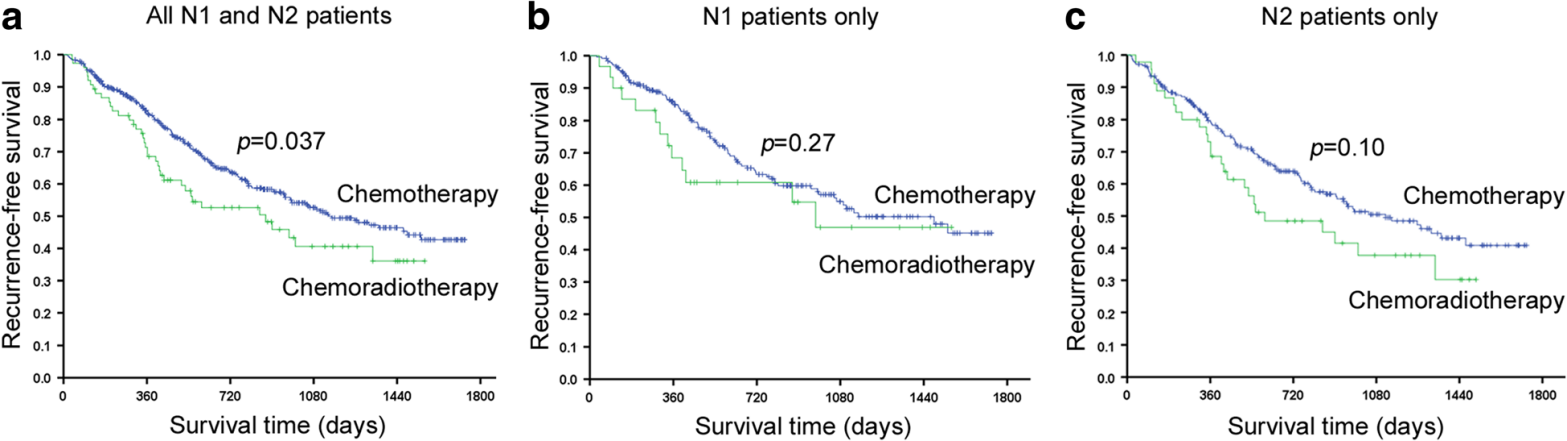
Kaplan–Meier curves for recurrence-free survival in postoperative SCLC patients receiving adjuvant chemotherapy or chemoradiotherapy. **a** Results for all N1 and N2 patients; **b** results for only N1 patients; and **c** results for only N2 patients

The Kaplan–Meier curves for recurrence-free survival are shown in Fig. 1. Recurrence-free survival was significantly longer in the chemotherapy compared with the chemoradiotherapy group, before adjusting for patient backgrounds (*P* = 0.037). However, this difference was not observed in separate analysis for each N1 (*P* = 0.27) and N2 group (*P* = 0.10).

The results of multivariable Cox regression analysis for recurrence-free survival are shown in Table 3. Recurrence-free survival did not differ significantly between the adjuvant chemoradiotherapy and chemotherapy groups (hazard ratio, 1.29; 95% CI, 0.91–1.84) after adjusting for age, sex, the extent of cancer (T and N factors), body mass index, Charlson comorbidity index, and Barthel index. Higher Charlson comorbidity index was significantly associated with shorter recurrence-free survival (hazard ratio, 1.81; 95% CI, 1.39–2.35).

**Table 3:** Multivariable Cox regression analysis of recurrence-free survival

	Hazard ratio	95% Confidence interval	*P*
Adjuvant therapy			
Chemotherapy	Reference		
Chemoradiotherapy	1.29	0.91–1.84	0.15
N factor			
N1	Reference		
N2	1.22	0.94–1.59	0.14
T factor			
T0-1	Reference		
T2	1.11	0.82–1.50	0.50
T3-4	1.09	0.77–1.56	0.63
Age (years)			
18–49	Reference		
50–64	1.27	0.62–2.59	0.51
65–74	1.27	0.64–2.55	0.50
≥ 75	1.46	0.69–3.09	0.33
Sex			
Male	Reference		
Female	0.77	0.54–1.10	0.15
Body mass index(kg/m^2^)			
< 18.5	1.42	0.87–2.31	0.16
18.5–24.9	Reference		
≥ 25	1.30	0.96–1.75	0.09
Charlson comorbidity index			
0–2	Reference		
≥ 3	1.81	1.39–2.35	<0.001
Activity of daily life (Barthel index)			
Independent (100–95)	Reference		
Dependent (≤90)	1.80	0.87–3.75	0.12

## Discussion

This study revealed no significant difference in prognosis between patients with SCLC diagnosed with N1-2 lymph node metastasis treated with postoperative adjuvant chemotherapy or chemoradiotherapy. To the best of our knowledge, this is the first study to provide evidence of treatment outcomes in these patients in a nationwide clinical setting.

An earlier study by the National Cancer Institute using information from the Surveillance, Epidemiology, and End Results database reported a median survival of 25.0 months in patients with stage II SCLC who underwent lung resection [6]. However, the current study had the advantage of including information not available in the previous study, including information on chemotherapy drugs, TNM classification, body mass index, Charlson comorbidity index, and Barthel index, thus allowing the prognostic effects of adjuvant chemotherapy and chemoradiotherapy to be compared after controlling for patient backgrounds. Patients with end-stage SCLC are often discharged to home care, hospices, nursing homes, or community hospitals. However, the DPC database only contains records of deaths occurring in-hospital, and we were therefore unable to follow-up all deaths outside the participating hospitals or home care. In the survival analyses of recurrence-free survival, discharge to another hospital or home was regarded as censored.

Some studies have suggested that resected patients receiving adjuvant chemotherapy had more favorable outcomes than non-resected patients receiving chemoradiotherapy [7, 8], possibly because surgery provides more effective local control than radiotherapy. The current study showed that adjuvant chemoradiotherapy had no clinical advantage over chemotherapy. Therefore, we were unable to demonstrate the treatment effect of additional radiotherapy in patients with SCLC who underwent lung resection followed by adjuvant chemotherapy. The lack of a significant difference in terms of recurrence-free survival suggests that chemotherapy alone may be preferable to adjuvant chemoradiotherapy, taking into consideration the potential adverse effects of chemoradiotherapy [9]. However, the present study was a retrospective, observational study, and further randomized clinical trials are needed to address this issue.

The present study also showed that some patients with SCLC received non-standard primary regimens in clinical practice. Although most patients received at least one cycle of chemotherapy containing cisplatin or carboplatin, some received neither etoposide nor irinotecan. This may have been because of the patient’s choice of chemotherapy drugs, or because of trials of new therapies for SCLC in some hospitals.

There were several limitations of this study associated with a lack of information on the pathological status of the dissected lymph nodes, the intent and detailed method of radiotherapy, and the severity of postoperative symptoms. Furthermore, the selection bias in choosing patients for adjuvant chemotherapy or chemoradiotherapy was unknown and might have affected the outcomes. Although it is generally assumed that surgery to remove one or more lung lobe(s) in patients with malignant lung cancer included complete lung resection and lymph node dissection, if more patients in the adjuvant chemoradiotherapy group had positive surgical margins or incomplete lymph node resection, this could have biased the results of our study against chemoradiotherapy. Furthermore, strong trends toward better outcomes in N2 patients in the adjuvant chemotherapy group may have been associated with the smaller number of patients with multiple N2, compared with the chemoradiotherapy group. Data on radiation field, dose, and intent of radiotherapy were also unavailable in the database. However, adjuvant thoracic irradiation usually comprises ≥15 daily fractions, and prophylactic cranial and other palliative irradiation usually involves no more than 10 daily fractions. We therefore defined adjuvant radiotherapy as radiation therapy lasting ≥17 consecutive days, within 5 months after surgery. This definition of adjuvant chemoradiotherapy was used to try and exclude most other palliative radiotherapies. The first course of chemotherapy or chemoradiotherapy for patients with SCLC is generally administered during hospitalization in Japan. Thus, most postoperative SCLC patients treated with adjuvant chemotherapy or chemoradiotherapy were likely to be included in our study. Patients may be followed up at the same hospital where they underwent lung resection, and may be admitted to the same hospital for examination and treatment of cancer relapse. This study may therefore have captured the initiation of chemotherapy or chemoradiotherapy and cancer relapse adequately, though lack of outpatient data may have biased the results.

Despite these limitations, recurrence-free survival in the adjuvant chemotherapy group in our study was similar to that in a previous prospective study [7], which also reported a 5-year survival of about 40% in stage II-IIIA SCLC patients with postoperative adjuvant chemotherapy.

## Conclusions

The present study showed no significant difference in recurrence-free survival between patients with SCLC and N1-2 lymph node metastasis treated with postoperative adjuvant chemotherapy and chemoradiotherapy. However, this was a retrospective study in relatively rare SCLC cases, and the possibilities of selection bias and unmeasured confounders mean that the results are inconclusive. Further clinical studies are thus needed to determine the optimal treatment strategy in patient with postoperative SCLC.

## Abbreviations

CI: Confidence interval
DPC: Diagnosis Procedure Combination
ICD-10: International Statistical Classification of Diseases and Related Health Problems-10th revision
SCLC: Small cell lung cancer
